# Supplementary material for: Identification of Autoantibodies to a Hybrid Insulin Peptide in Type 1 Diabetes
Source: Diagnostics (Basel). 2023 Sep 4;13(17):2859. doi: 10.3390/diagnostics13172859 (PMC10487141; doi:10.3390/diagnostics13172859)
Supplement: Supplementary file 1 [file diagnostics-13-02859-s001.zip › diagnostics-2579716-supplementary.pdf]

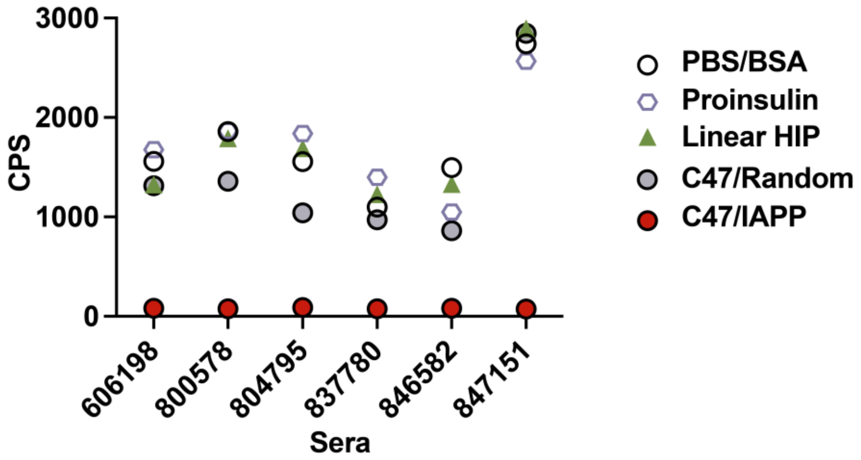

Supplementary Figure S1. ***Investigation of pre-absorption of C47/IAPP HIP antibodies using unlabeled Linear HIP or C47/Random probes.*** *Sera was preincubated with the indicated proteins prior to ECL assay. Graph showing binding of T1D patient sera to labeled C47/IAPP after pre-absorption with C47/IAPP (solid red circles), linear HIP (green closed triangles), proinsulin (gray open circles), C47/Random probe (gray closed circles) or non-specific (PBS) control (black open circles). C47/Random is an identical probe to C47/IAPP with the IAPP amino acids in reverse sequence.*
